# Thyroid hormone-mediated regulation of lipocalin 2 through the Met/FAK pathway in liver cancer

**DOI:** 10.18632/oncotarget.3670

**Published:** 2015-04-10

**Authors:** I-Hsiao Chung, Cheng-Yi Chen, Yang-Hsiang Lin, Hsiang-Cheng Chi, Ya-Hui Huang, Pei-Ju Tai, Chia-Jung Liao, Chung-Ying Tsai, Syuan-Ling Lin, Meng-Han Wu, Ching-Ying Chen, Kwang-Huei Lin

**Affiliations:** ^1^ Department of Biochemistry, School of Medicine, Chang-Gung University, Taoyuan, Taiwan; ^2^ Department of Medical Research, Mackay Memorial Hospital, Taipei, Taiwan; ^3^ Liver Research Center, Department of Hepato-Gastroenterology, Chang-Gung Memorial Hospital, Linkou, Taoyuan, Taiwan

**Keywords:** thyroid hormone receptor, LCN2, Met/FAK cascade

## Abstract

The thyroid hormone, 3,3′,5-triiodo-L-thyronine (T_3_), regulates cell growth, development and differentiation via interactions with thyroid hormone receptors (TR), but the mechanisms underlying T_3_-mediated modulation of cancer progression are currently unclear. Lipocalin 2 (LCN2), a tumor-associated protein, is overexpressed in a variety of cancer types. Oligonucleotide microarray, coupled with proteomic analysis, has revealed that LCN2 is positively regulated by T_3_/TR. However, the physiological role and pathway of T_3_-mediated regulation of LCN2 in hepatocellular carcinogenesis remain to be characterized. Upregulation of LCN2 after T_3_ stimulation was observed in a time- and dose-dependent manner. Additionally, TRE on the LCN2 promoter was identified at positions −1444/−1427. Overexpression of LCN2 enhanced tumor cell migration and invasion, and conversely, its knockdown suppressed migration and invasion, both *in vitro* and *in vivo*. LCN2-induced migration occurred through activation of the Met/FAK cascade. LCN2 was overexpressed in clinical hepatocellular carcinoma (HCC) patients, compared with normal subjects, and positively correlated with TRα levels. Both TRα and LCN2 showed similar expression patterns in relation to survival rate, tumor grade, tumor stage and vascular invasion. Our findings collectively support a potential role of T_3_/TR in cancer progression through regulation of LCN2 via the Met/FAK cascade. LCN2 may thus be effectively utilized as a novel marker and therapeutic target in HCC.

## INTRODUCTION

The thyroid hormone (TH) regulates cell growth, development and differentiation by binding to thyroid hormone receptors (TR), which belong to a superfamily of nuclear receptors. Human TRs are encoded by TRα and TRβ genes located on human chromosomes 17 and 3, respectively [1]. These receptors function as ligand-dependent transcription factors that form heterodimers with the retinoid X receptor (RXR) and regulate target genes via binding to thyroid hormone response elements (TRE) located in their promoter regions [2]. In particular, TRs bind to TREs in which half-sites are arranged as palindromes (Pal), direct repeats (DR), and inverted palindromes (IPs). T_3_ is implicated as a potential tumor inducer in several cancer types [3–5]. The T_3_/TR complex promotes intestinal cell proliferation and intestinal tumorigenesis via cooperation with the WNT pathway, and induces β-catenin and some of its targets [6]. Additionally, hyperthyroxinemia is reported to increase the rate of colon cancer incidence in a rat experimental model [7]. The findings to date collectively implicate a critical role of T_3_/TR signaling in tumorigenesis.

Previous microarray analysis, coupled with proteomic data, revealed the existence of a 25 kDa protein regulated by T_3_, lipocalin 2 (LCN2), which belongs to the lipocalin gene superfamily located at human chromosome 9q34. Members of the superfamily are composed of 20 small secreted lipoproteins that interact with specific ligands and share the same three-dimensional structure in an eight-stranded anti-parallel β-barrel surrounding a central pocket. The most important domain of LCN2 is the central calyx, which is responsible for its activity as a transporter of small hydrophobic substances, such as prostaglandins, retinoids, arachidonic acid and fatty acids [8]. LCN2 is currently one of the most interesting and enigmatic proteins involved in the process of tumor development [9].

Increased LCN2 expression has been described in variety of cancers [10]. However, the specific functions of LCN2 and mechanisms underlying its modulation of cancer progression are not well characterized at present. An association between LCN2 and tumor invasion/metastasis is documented [11]. Recent studies demonstrated that LCN2 induces epithelial-mesenchymal transition (EMT) and upregulation of MMP-9 in cancer progression [12, 13]. LCN2 is a recognized target gene of T_3_/TR signaling, which enhances cell metastasis [14]. However, the mechanistic pathway linking T_3_/TR with LCN2 function in metastasis has not been characterized as yet. Here, we focused on the role of LCN2 and the mechanism underlying its regulation by T_3_/TR in a hepatoma cell line. Based on the collective findings, we propose that T_3_/TR promotes metastasis through LCN2 upregulation in HCC.

## MATERIALS AND METHODS

### Cell culture

Human hepatoma cells, HepG2, Huh7, SK-HEP1 and J7, were routinely cultured at 37°C in a humidified atmosphere of 95% air and 5% CO_2_ in Dulbecco's modified Eagle's medium (DMEM) supplemented with 10% fetal bovine serum (FBS). HepG2 and J7 cell lines were stably transfected with TRα1 (HepG2-TRα1 and J7-TRα1) or TRβ1 (HepG2-TRβ1). The vector control cell line employed was HepG2-neo [15, 16] Huh7-LCN2 and SK-HEP1-LCN2 represent Huh7 and J7 cell lines expressing LCN2, respectively. Serum was depleted of T_3_ (Td), as described previously [17].

### Preparation of conditioned medium

HepG2-TRα1, HepG2-TRβ1, HepG2-neo, J7-TRα1, Huh7-LCN2 and SK-HEP1-LCN2 cells were grown to confluence in 10 cm cell culture dishes. Cells contacting dishes were washed twice with PBS, subsequently incubated in serum-free medium, and either treated with T_3_ for 24 h or left untreated. At the end of the treatment period, conditioned medium (CM) was collected and concentrated using spin columns with a molecular mass cut-off of 3 kDa (Amicon Ultra, Millipore, Billerica, MA).

### Immunoblot analysis

Total cell lysates and conditioned media were prepared, and protein concentrations determined with the Bradford assay kit (Pierce Biotechnology, Rockford, IL). Equivalent amounts of proteins were fractionated on a 10% sodium dodecyl sulfate (SDS)-polyacrylamide gel. Separated proteins were transferred to nitrocellulose membrane (pH 7.9, Amersham Biosciences Inc., Piscataway, NJ), blocked with 5% non-fat powdered milk, incubated with specific primary antibodies at 4°C overnight, and subsequently hybridized with the respective secondary antibody (HRP-conjugated mouse/rabbit/goat anti-IgG) for 1 h at room temperature. Finally, immune complexes were visualized using the chemiluminescence method with an ECL detection kit (Amersham) on Fuji X-ray film, as described previously [18].

### Quantitative reverse transcription polymerase chain reaction (q-RT-PCR)

Total RNA was extracted from four T_3_-treated HepG2 isogenic cell lines using TRIzol reagent, as described previously [19]. Subsequently, cDNA was synthesized via RT-PCR with the Superscript II kit (Life Technologies, Karlsruhe, Germany). Real-time qRT-PCR was performed on a 15 μl reaction mixture containing 750 nM forward and reverse primers, varying amounts of template and 1 × SYBR Green reaction mix (Applied Biosystems, Foster City, CA). SYBR Green fluorescence was determined using the ABI PRISM 7500 detection system (Applied Biosystems). Primers were designed using Primer Express Software (Applied Biosystems). Genes were normalized against the ribosomal binding protein *RiboL35A* gene.

### Cloning and activities of LCN2 promoter fragments

Fragments of the *LCN2* promoter (positions −1524 to +98) were ligated into the pA3TK vector (Promega Corp., Madison, WI) based on the published sequence. Several serial deletion and mutant constructs of the *LCN2* promoter were amplified via PCR and cloned into pA3TK. Promoter sequences were confirmed using automated DNA sequencing. HepG2-TRα1 cells treated with 10 nM T_3_ for 24 h were cotransfected with 0.6 μg DNA/well of pA3TK vector containing the *LCN2* promoter sequence and 0.3 μg of SVβ plasmid, a β-galactosidase expression vector (Clontech, Palo Alto, CA), in 24-well plates using TurboFect *in vitro* transfection reagent (Fermentas, Glen Burnie, MD) to determine the transcriptional activities of TREs within the *LCN2* promoter. At the end of the treatment period, transfected and non-transfected cells were lysed, and the luciferase and β-galactosidase activities measured. Luciferase activity was normalized to that of β-galactosidase, as described earlier [20].

### Chromatin immunoprecipitation (ChIP) assay

ChIP assays were performed to examine the interactions between TR and TRE on the *LCN2* promoter [18]. HepG2-TRα1 cells treated with 10 nM T_3_ for 24 h or left untreated were harvested and cross-linked with 1% formaldehyde for 10 min at room temperature in DMEM. Reactions were terminated with the addition of 0.125 M glycine. Subsequently, cell lysates were washed three times with PBS and resuspended in lysis buffer (150 mM NaCl, 5 mM EDTA, 50 mM Tris (pH 8.0), 0.1% SDS and 0.1% sodium deoxycholate) containing three protease inhibitors (1 mM PMSF, aprotinin, and leupeptin). Cell lysates were sonicated with a Misonix Sonicator 3000 Homogenizer (Mandel Scientific Company Inc., Guelph, ON, Canada) to disrupt chromatin. Sonicated DNA was between 200 and 1000 bp in length. Products were precleared with 60 μl protein A/G agarose (Sigma Chemicals, St. Louis, MO) for 2 h at 4°C. Complexes were immunoprecipitated with anti-TR (kindly provided by the laboratory of Dr. S-Y Cheng at the National Cancer Institute) and anti-IgG antibodies (R&D Systems, Inc., Minneapolis, MN). The 59 bp *LCN2* promoter fragment containing the predicted TRE region was amplified via PCR with the forward primer, 5′- TCAGGTACCCGGCCTGGCAGAGGATAC-3′, and reverse primer, 5′-TCACTC GAGCCCAGGAACTCCACCTCTG-3′.

### Cloning of LCN2

Total RNA (1 μg) was reverse-transcribed using Superscript II reverse transcriptase (Invitrogen) and Oligo (dT) to synthesize template cDNA. *LCN2* cDNA was amplified via PCR with the forward primer, 5′-TCAGGTACCATGC CCCTAGGTCTCCTGTG-3′, and reverse primer, 5′-CTCCTCGAGTCAGCCGT CGATACACTGGT-3′, for 30 cycles at 95°C for 1 min, 58°C for 1 min and 72°C for 2 min. The *LCN2* open reading frame was ligated into pcDNA 3.0 expression vector, and the resulting construct sequenced to confirm the presence of the gene.

### Establishing Huh7 and SK-HEP1 cell lines stably overexpressing LCN2

Huh7 and SK-HEP1 cell lines were transfected with the LCN2 cDNA construct in 10 cm cell culture dishes using Lipofectamine Reagent (Invitrogen). After 24 h, transfected cells were transferred to medium containing G418 (400 μg/ml) for selection until the generation of a single cell clone. Expression of LCN2 protein in Huh7 and SK-HEP1 cells was detected using Western blot analysis.

### Effects of knockdown of LCN2 expression

Short hairpin RNA clones targeting LCN2 were purchased from the National RNAi Core Facility (Institute of Molecular Biology, Academia Sinica, Taiwan). Transfection of shRNA against the endogenous *LCN2* gene in HepG2-TRα1 and J7 cells was transit performed using Turbofect reagent (Invitrogen). LCN2 repression was confirmed via Western blot analysis.

### *In vitro* migration and invasion assays

The influence of LCN2 on the migration and invasion abilities of Huh7-LCN2 and SK-HEP1-LCN2 cells was determined with a rapid *in vitro* assay (Transwell) (Falcon BD, Franklin Lakes, New Jersey) [21]. Briefly, cell density was adjusted to 10^5^ cells/ml, and 100 μl of the suspension seeded on either non-matrigel-coated (migration) or matrigel-coated (invasion) (Becton-Dickinson) upper chambers of the Transwell plate. For both assays, the pore size of the upper chamber was 8 mm. The medium in the upper chamber was serum-free DMEM, while the lower chamber contained DMEM supplemented with 20% fetal bovine serum (FBS). After incubation for 24 h at 37°C, cells traversing the filter from the upper to lower chamber were examined via crystal violet staining and cell counting. Experiments were performed at least three times.

### Immunohistochemistry staining

Formalin-fixed and paraffin-embedded tissues from lungs of SCID mice were evaluated via hematoxylin and eosin (H&E) staining and immunohistochemistry using a polyclonal antibody against LCN2 (GeneTex, Inc, San Antonio, Texas) after the avidin-biotin complex method, as described previously. Positive staining of cancer cells was identified as dark brown color indicative of LCN2 immunoreactivity.

### Gelatin zymography

Supernatant fractions of J7-control and J7-LCN KD cells cultured for 24 h were collected and concentrated using Amicon Ultra-4 Centrifugal Filter Devices (Merck Millipore Ltd.). Equal amounts of proteins were separated via 10% SDS-PAGE with 0.1% gelatin (Sigma). The gel was incubated in reaction buffer (40 mM Tris-HCl, pH 8, 10 mM CaCl_2_, and 1% NaN_3_) at 37ºC overnight, stained with 0.25% Coomassie Brilliant Blue R-250 in 10% acetic acid and 50% methanol for 30 min, and de-stained with 10% acetic acid and 20% methanol twice for 30 min.

### Animals

Similar conditions were employed with SCID mice containing various T_3_ levels induced via injection of J7-TR cells [14]. Mice were divided into two groups, specifically, Group A (euthyroid) comprising control mice given normal drinking water and Group B (hyperthyroid) administered drinking water augmented with T_3_ (2 mg/L) (Sigma Chem. Co., St. Louis, MO) after inoculation of tumor cells. Mice were sacrificed about 1 month after injection, their livers and lungs removed for tumor biopsy, and the T_3_ and TSH levels determined. The T_3_ and TSH levels in sera of euthyroid mice (Group A) were 45.5 ng/dl and 0.246 mIU/ml, while those in sera of hyperthyroid mice (Group B) were 619 ng/dl and 0.008 mIU/ml, respectively. Tumor volume was calculated using the following equation: length × height × width. Formalin-fixed and paraffin-embedded tissues from SCID mice were evaluated based on hematoxylin and eosin (H&E) staining and immunohistochemistry using a polyclonal antibody against LCN2 (Epitomics). All procedures were performed under sterile conditions in a laminar flow hood. Animal experiments were performed in accordance with the United States National Institutes of Health guidelines and Chang-Gung Institutional Animal Care and Use Committee Guide for the Care and Use of Laboratory Animals.

### Human HCC specimens

All samples of HCC tissues with paired adjacent normal liver tissues for Western blot and q-RT-PCR analyses were from the Taiwan Liver Cancer Network (TLCN). The study protocol was approved by the Medical Ethics and Human Clinical Trial Committee of the Chang Gung Memorial Hospital (IRB No. 98–0798B).

### Statistical analysis

Data are expressed as mean values ± SEM of at least three experiments. Statistical analysis was performed using Student's *t* test and one-way ANOVA. *P* values < 0.05 were considered statistically significant.

## RESULTS

### Effects of T_3_ on LCN2 mRNA and protein expression

**Figure 1:**
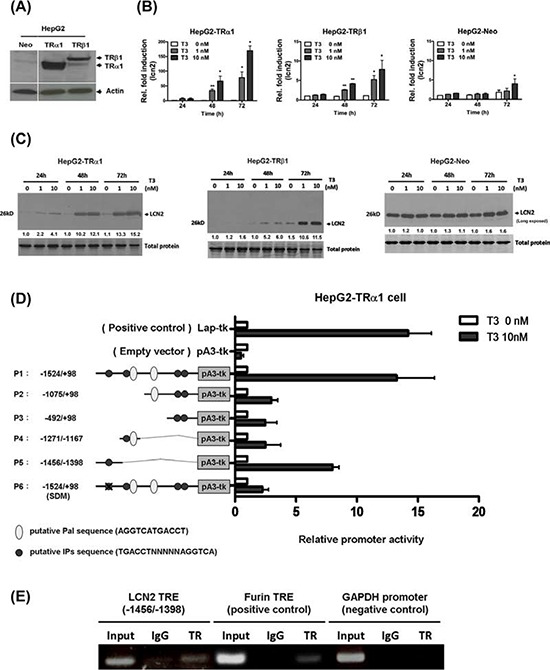
T_3_ regulates LCN2 mRNA and protein expression in HepG2 cells **A.** Expression of TR in extracts of TR-overexpressing and parental cell lines was determined via Western blot analysis. The positions of 47 kDa TRα1 and 55 kDa TRβ1 are indicated. LCN2 mRNA and protein expression was determined in three stable HepG2-TR and HepG2-neo cell lines at 24–72 h in the absence or presence of 1 and 10 nM T_3_ using **B.** q-RT-PCR and **C.** Western blot, respectively. **D.** HepG2-TRα1 cells were transfected with a luciferase reporter plasmid driven by the minimum thymidine kinase promoter and including the *LCN2* 5′-flanking region (positions −1524 to +98 encompassing six putative TRE sites) with or without T_3_. Promoter activities were calculated relative to 10 nM T_3_ (+T_3_/−T_3_), and further normalized to pA3TK-luc control as well as β-galactosidase activity (T_3_-induced changes were normalized to that of β-gal). Columns, mean values obtained from at least three independent experiments performed in triplicate; bars, SE. SDM: *site*-*directed mutagenesis* **E.** ChIP assay demonstrating that TR is recruited to the *LCN2* 5′-flanking region. Two sets of primers for *LCN2* TRE, positive control TRE (*FURIN*) and negative control (*GAPDH*), were prepared. Differences were analyzed using One-way ANOVA, *P < 0.05.

Several isogenic HepG2 cell lines stably expressing high levels of wild-type TRα1 and TRβ1 (HepG2-TRα1 and HepG2-TRβ1, respectively) were established. Three HepG2 cell lines, HepG2-Neo, HepG2-TRα1 and HepG2-TRβ1, expressing various levels of TR (Fig. 1A) were used for analyses. Notably, LCN2 expression was stimulated by T_3_ in HepG2-TRα1 and HepG2-TRβ1 cell lines at both the mRNA (Fig. 1B) and protein levels (Fig. 1C) in a time- and dose-dependent manner. In contrast, T_3_ had a marginal or no effect on LCN2 expression in HepG2-Neo cells (Fig. 1B, C), suggesting that LCN2 is stimulated by T_3_ in a receptor-dependent manner.

### T_3_ induces LCN2 transcription

The reporter assay was performed to identify the position of the thyroid hormone response element (TRE) to further clarify the regulatory effects of T_3_ on *LCN2* at the transcriptional level. The *LCN2* 5′-flanking region encompassing nucleotides −1524/−98 (relative to the transcription initiation site) with numerous predicted putative TREs (Fig. 1D) was cloned and inserted upstream of the luciferase reporter gene in pA3TK-luc containing a minimum thymidine kinase promoter to generate Construct p1. Serial deletion mutants were subsequently generated, designated Constructs p2–5 (Fig. 1D). The transcriptional activities of the *LCN2* promoter fragments are illustrated in Fig. 1D. Among these, only the p5 construct containing one putative TRE was activated ~8-fold by T_3_ in HepG2-TRα1 cells. TREs in the p1 fragment were sequentially mutated to yield p6 constructs. Upon mutation of the putative TRE (IPs), the luciferase activity of p6 was completely abolished (Fig. 1D). These findings suggest that T_3_ regulates *LCN2* at the transcriptional level by binding to the putative TRE site between positions −1456/−1398 (p5) encompassing an IP-like sequence between positions −1444 and −1427 (GGATACTTTTTAAGGTCA).

### TR proteins interact with TRE (positions −1444 to −1427) within the LCN2 promoter

To further determine whether LCN2 TRE (IPs) is directly targeted by TR proteins, the ChIP assay was performed. TR proteins were clearly associated with the TRE region within the *LCN2* promoter *in vivo* (Fig. 1E). Notably, TRα1 was recruited to the TRE-binding site whereas control IgG produced only background levels (Fig. 1E). Furin TRE was used as the positive control [15]. Accordingly, we propose that TRα1 protein binds the *LCN2* promoter for transcriptional regulation.

### LCN2 is associated with cancer progression *in vitro* and *in vivo*

**Figure 2:**
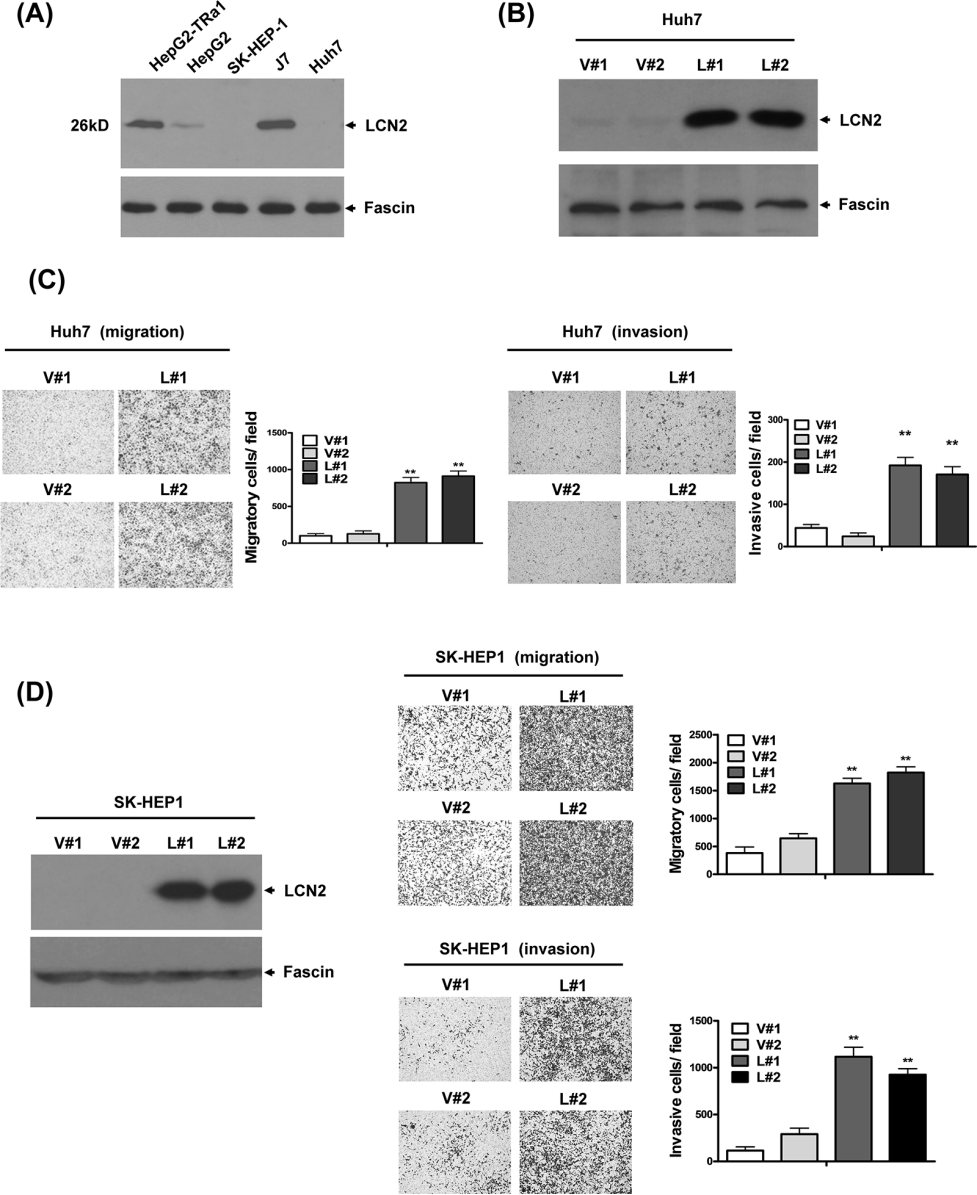
LCN2 promotes cell migration and invasion *in vitro* **A.** Endogenous LCN2 expression was analyzed in various hepatoma cell lines via Western blot. **B.** Expression of LCN2 in Huh7 cells was detected in LCN2-overexpressing clones (L#1, L#2) and controls (V#1, V#2) using Western blot. **C.** Migration ability was analyzed in two LCN2-overexpressing and two control Huh7 cell lines using the Transwell assay. The number of cells traversing the filter to the lower chamber was counted to determine migration activity. Transwell filters were stained with crystal violet in the left panel, and migration ability quantified in the right panel. **D.** Expression of LCN2 in SK-HEP1-control (V#1, V#2) and SK-HEP1-LCN2 (L#1, L#2) cells was determined using Western blot (left panel). Migration and invasion abilities were examined with the Transwell assay. Differences were analyzed using One-way ANOVA, *P < 0.05.

LCN2 expression was identified in five hepatoma cell lines (Fig. 2A). To determine the specific function of LCN2, control cell lines Huh7-V#1 and V#2 (Fig. 2B) or SK-HEP1-V#1 and V#2 (Fig. 2D) and those overexpressing LCN2, Huh7-L#1 and L#2 (Fig. 2B) or SK-HEP1-L#1 and L#2 (Fig. 2D), were established. Notably, Huh7 cell lines overexpressing LCN2 displayed significantly increased (~4 to 5-fold) migration and (~3 to 4-fold) invasion, compared with control cells (Fig. 2C). Similarly, we observed markedly increased (~3 to 5-fold) migration and (~4 to 5-fold) invasion in the SK-HEP1 cell lines overexpressing LCN2 (Fig. 2D). Our results indicate that LCN2 functions in cell migration and invasion, but has no effect on cell growth (data not shown).

**Figure 3:**
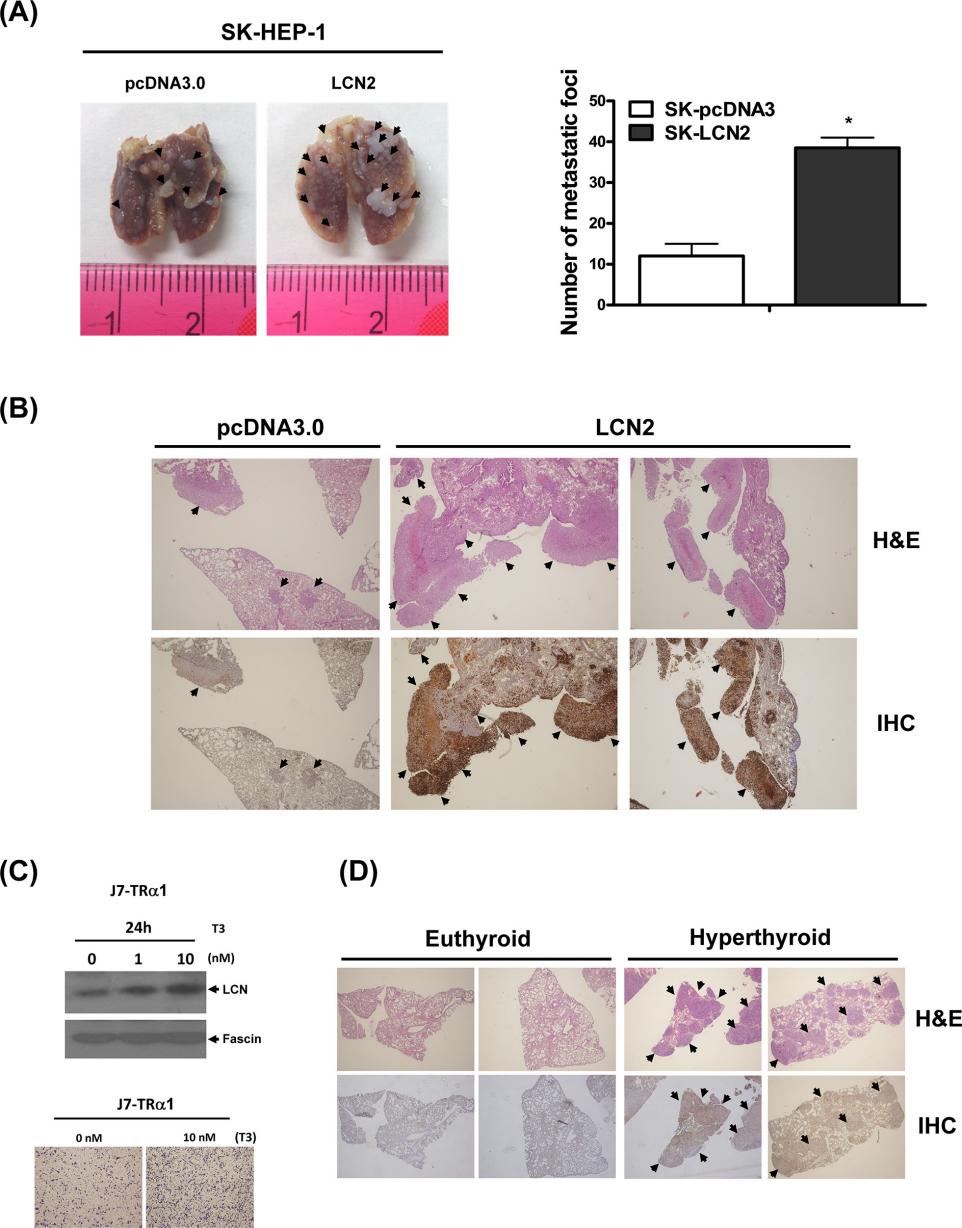
LCN2 promotes cell migration and invasion *in vivo* (**A**. left panel) Images depict lung tumor foci of SK-HEP1-control and SK-HEP1-LCN2 cells. (A, right panel) Metastatic foci in lung were quantified. **B.** Tumor foci and LCN2 expression of SK-HEP1-control (B, left panel) and SK-HEP1-LCN2 cells (B, right panel) were examined using H&E staining (B, upper panel) and IHC (B, lower panel), respectively. (**C**, upper panel) LCN2 expression in J7-TRα1 cells treated with T_3_ was detected via Western blot. (C, lower panel) Migration ability was determined with the Transwell assay. **D.** Lung sections of SCID mice injected with J7-TRα1 cells treated with high levels of T_3_ (hyperthyroid) and administered normal drinking water (euthyroid) were analyzed with H&E (upper panel) and IHC (lower panel) staining to examine LCN2 expression and tumor foci. Differences were analyzed using One-way ANOVA, *P < 0.05.

To verify whether the *in vitro* effect of LCN2 can be replicated *in vivo*, SCID mice were injected with SK-HEP1-LCN2 (LCN2-pooled stable clone) and SK-HEP1-control (pcDNA3.0-pooled stable clone) cells. Significantly, SK-HEP1-LCN2 cells formed higher numbers of lung foci in SCID mice (Fig. 3A), compared with control cells, and displayed elevated LCN2 expression, as evident from H&E staining and IHC analyses (Fig. 3B), respectively. Thus, LCN2 appears to promote cell migration and invasion in SK-HEP1 hepatoma cells, both *in vitro* and *in vivo*.

To further investigate the T_3_-regulated effects *in vitro* and *in vivo*, we established a J7-TRα1 cell line. LCN2 protein expression was stimulated by T_3_ in J7-TRα1 cells (Fig. 3C, upper panel) in a dose-dependent manner. Consistently, the migration ability of J7-TRα1 cells was significantly enhanced upon T_3_ stimulation (Fig. 3C, lower panel). SCID mice were injected with J7-TRα1 cells and subjected to several T_3_ conditions [14]. Mice injected with J7-TRα1 cells displayed multiple macroscopic lung tumor nodules, determined with hematoxylin and eosin (H&E) staining. Higher T_3_ levels (hyperthyroid conditions) led to enhanced LCN2 expression and number of lung foci, as evident from H&E staining and IHC (Fig. 3D, S3A and S5). LCN2 expression in metastatic lung foci of hyperthyroid mice was obviously higher than that in euthyroid mice (normal T_3_ levels). Moreover, we observed T_3_-induced cancer cell invasion and LCN2 expression *in vivo*, supporting the theory that T_3_ influences tumor motility via LCN2 regulation.

### LCN2 depletion suppresses cancer progression *in vitro* and *in vivo*

**Figure 4:**
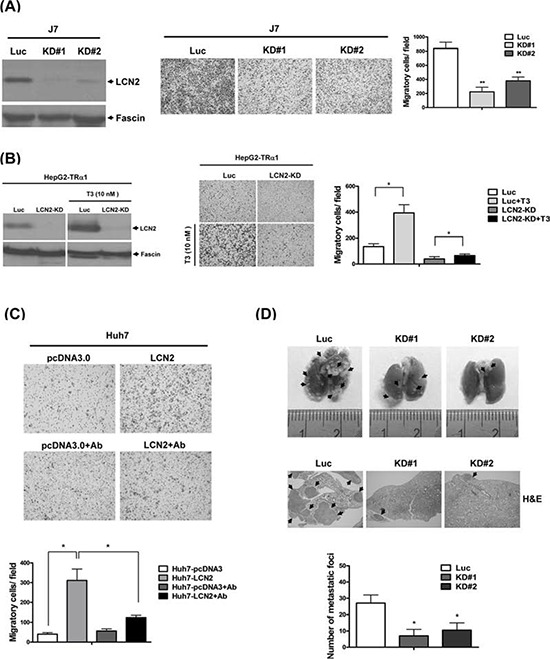
LCN2 depletion suppresses cell mobility *in vitro* and *in vivo* **A.** Expression of LCN2 in J7 cells was detected in LCN2-depleted clones (KD#1, KD#2) and controls (Luc) via Western blot (left panel). Migration ability was analyzed in two LCN2-depleted and control J7 cell lines using a Transwell assay (middle panel). Quantified results are shown (right panel). **B.** LCN2 expression and depletion in HepG2-TRα1 control (Luc) and HepG2-TRα1-LCN2 knockdown (LCN2-KD) cells treated with T_3_ was detected via Western blot analysis (left panel). Migration ability was determined using the Transwell assay (middle panel), and data quantified (right panel). **C.** Application of the LCN2-neutralizing antibody in Huh7 LCN2-overexpressing and Huh7 pcDNA3.0 cells (C, upper panel), compared to treatment with IgG antibody (C, upper panel), and quantification of migration ability (lower panel). **D.** The images depict lung tumor foci of mice administered J7-control (Luc) and J7-LCN2-knockdown cells (KD#1 and KD#2) (upper panel). The number of metastatic foci in lung for J7-control and J7-LCN2-knockdown cell groups is specified (lower panel). Tumor foci of J7-control and J7-LCN2-knockdown cells were examined via H&E staining (middle panel).

To determine the consequences of LCN2 depletion, control cell lines J7-Luc (Fig. 4A, left panel) or HepG2-TRα1-Luc (Fig. 4B, left panel) and LCN2 knockdown lines, J7-KD#1 and KD#2 (Fig. 4A) or HepG2-TRα1-LCN2-KD (Fig. 4B) were established. Data from the transwell assay showed that after depletion of LCN2 in J7 or HepG2-TRα1 cells, migration abilities were decreased, compared with those of control cells (Fig. 4A, B, middle panel), confirming the ability of LCN2 to accelerate tumor cell migration *in vitro*. LCN2 expression in control cells was induced by T_3_ to further confirm the thyroid hormone effect. Induction of expression was abolished in HepG2-TRα1-LCN2-KD cells (Fig. 4B, middle panel). Moreover, the migration ability of HepG2-TRα1-control cells was markedly enhanced following T_3_ stimulation, but attenuated in HepG2-TRα1-LCN2-KD cells (Fig. 4B, middle panel). Notably, migration was minimally restored in the HepG2-TRα1-LCN2-KD-T_3_ cell line after T_3_ treatment, compared to the LCN2-silenced HepG2-TRα1-LCN2-KD cell line. Migration was blocked by ~80% in HepG2-TRα1-LCN2-KD cells treated with T_3_, compared with HepG2-TRα1-control cells (Fig. 4B, middle and right panels). Treatment with a LCN2-neutralizing antibody additionally induced a significant decrease in migration (~3-fold) in Huh7-LCN2, but not control cells (Fig. 4C). Taken together, the data indicate that the migration phenotype in the T_3_-treated hepatoma cells can be restored in LCN2-KD conditions, but attenuated by a LCN2-neutralizing antibody.

To investigate the effects of LCN2 depletion *in vivo*, SCID mice were injected with J7-LCN2-KD (LCN2-pooled KD stable clone) and J7-control (Luc-pooled stable clone) cells. Mice administered J7-LCN2-KD cells displayed significantly reduced numbers of lung foci (Fig. 4D, upper panel), compared to those injected with control cells, as evident from H&E staining results (Fig. 4D, middle panel). Accordingly, we conclude that LCN2 depletion reduces migration and invasion in J7 hepatoma cells, both *in vitro* and *in vivo*.

### LCN2 regulates cancer-related molecules

**Figure 5:**
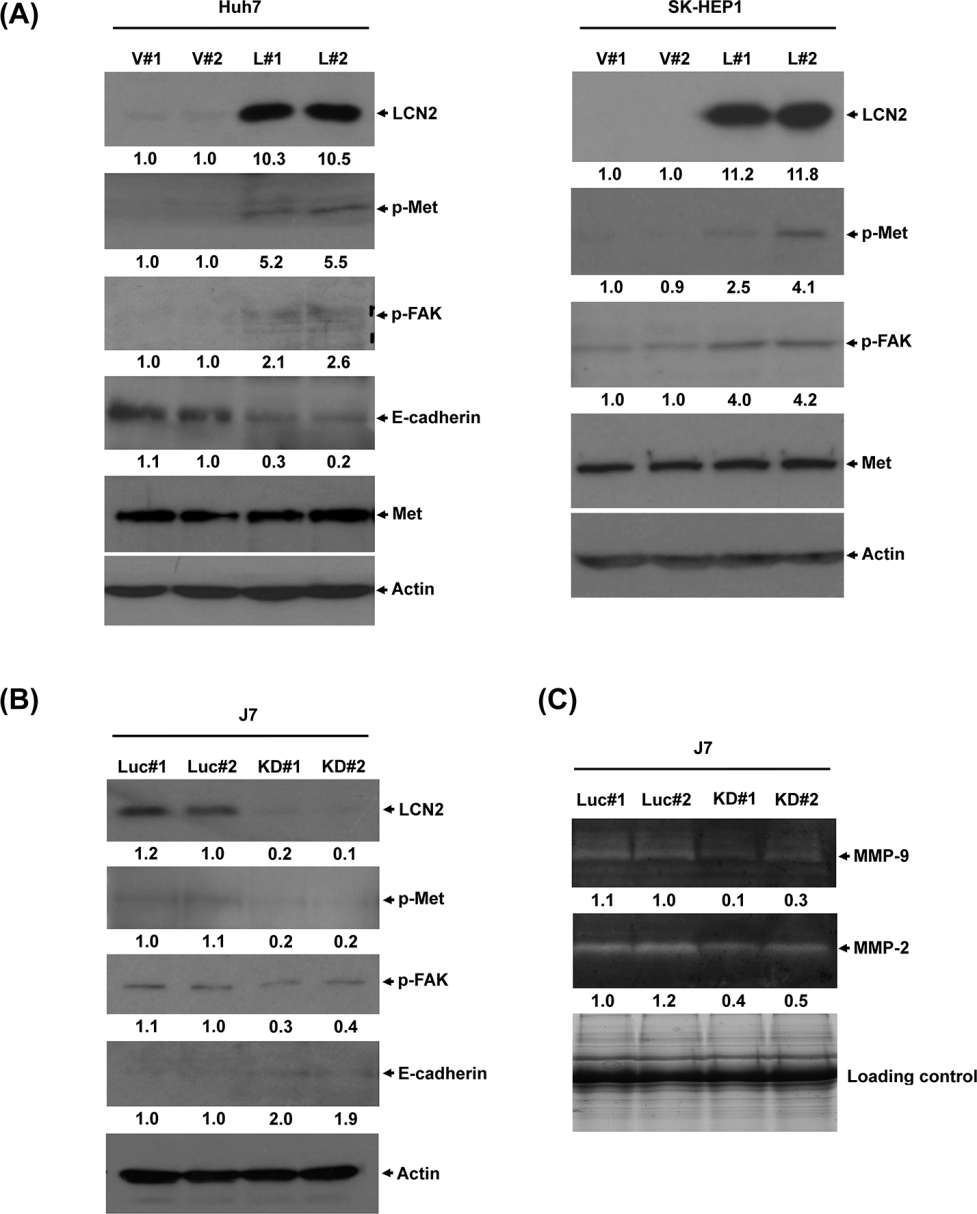
LCN2 regulates cell mobility through the Met/FAK cascade **A.** Expression of phosphorylated Met and FAK and E-cadherin in Huh7 and SK-HEP1 LCN2-overexpressing (L#1, L#2) and control (V#1, V#2) or **B.** J7 LCN2 knockdown (KD#1, KD#2) and control cells. **C.** MMP-9 and MMP-2 activities in J7 LCN2 knockdown cells.

LCN2 is associated with EMT, and promotes cancer cell migration [11]. The proto-oncogene c-Met is a key regulator of EMT-induced cell migration and invasion [22]. Earlier, Chen *et al*. [23] demonstrated that cellular migration is mediated downstream of c-Met through its phosphorylation of focal adhesion kinase (FAK). Accordingly, we examined whether the Met/FAK activation pathway is implicated in LCN2-induced phenotypes. Marked upregulation of p-Met and p-FAK was observed in LCN2-overexpressing cells (Huh7-LCN2, L#1 and L#2 ; SK-HEP1-LCN2, L#1 and L#2), compared with control cells (Huh7-control, V#1 and V#2; SK-HEP1-control, V#1 and V#2) (Fig. 5A). Furthermore, after depletion of LCN2 in J7, p-Met and p-FAK protein levels, MMP-9 and MMP-2 activities were decreased, compared with control cells (Fig. 5B, C). Further examination of whether the LCN2-mediated mechanism occurs in T_3_-regulated hepatoma cells revealed upregulation of p-Met and p-FAK after T_3_ stimulation in HepG2-TRα1 cells (Fig. S1). Based on the collective findings, we conclude that stimulation of p-Met and p-FAK by T_3_ in HepG2-TRα1 cells is mediated via LCN2.

### LCN2 is upregulated in human HCC

**Figure 6:**
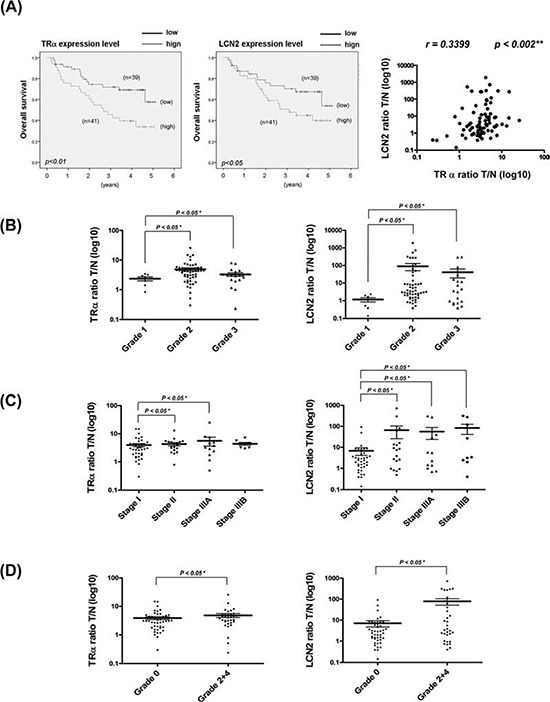
Clinical correlations between TR and LCN2 and associated parameters in HCC **A.** Expression of LCN2 and TR mRNA in 80 paired HCC specimens determined using q-RT-PCR. Overall survival rates of TR and LCN2 were analyzed with SPSS software. The blue line indicates low expression of TR and LCN2, and the green line represents high levels of TR (left) and LCN2 (right). Correlations of T/N ratios between TR and LCN2 (right panel) were analyzed using linear regression. In relation to **B.** tumor grade, **C.** TNM stage, and **D.** vascular invasion grade, the T/N ratios of TR and LCN2 showed similar patterns. Differences were analyzed using One-way ANOVA, *P < 0.05.

**Figure 7:**
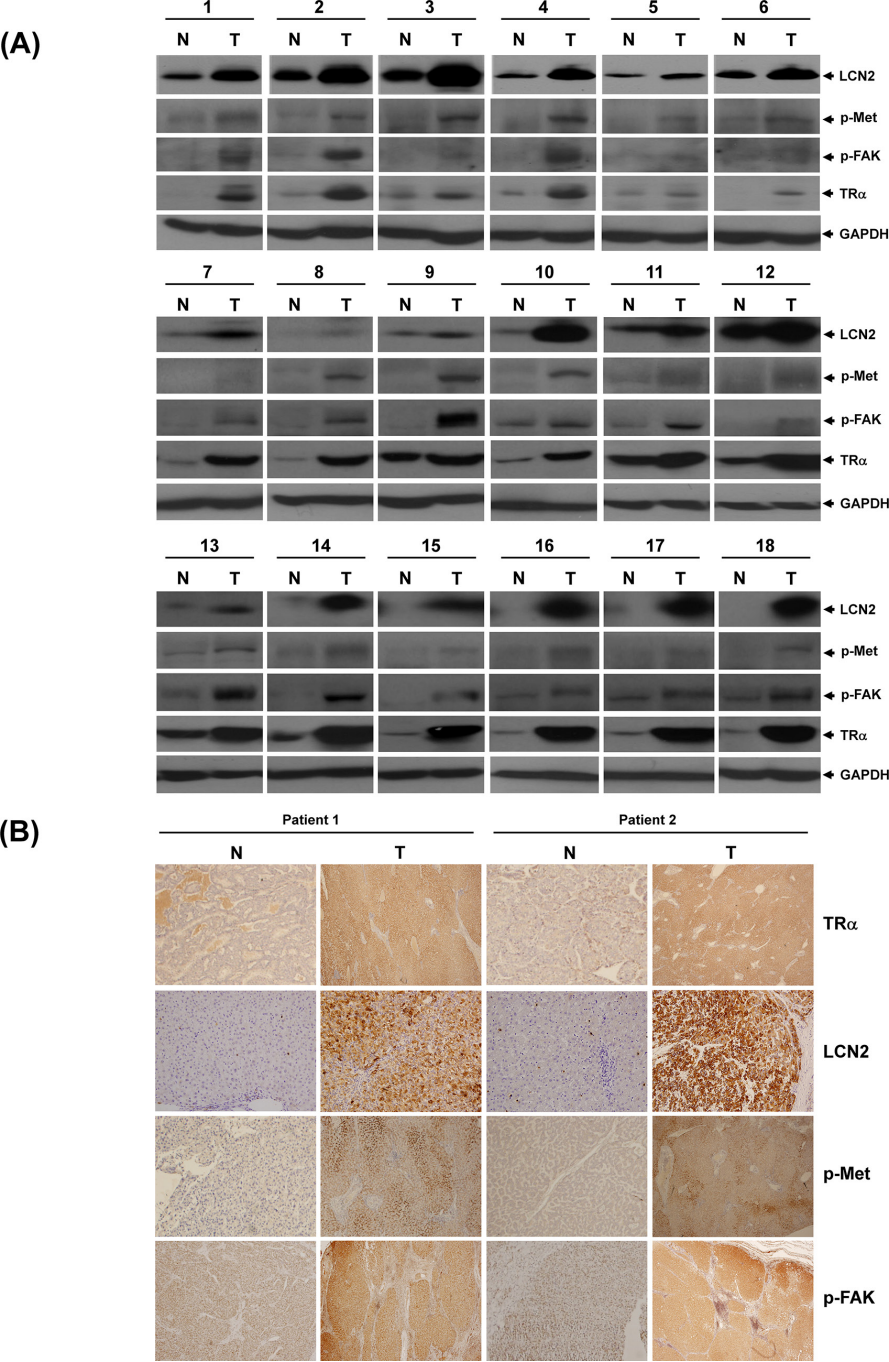
LCN2 and TR expression in clinical specimens TR, LCN2, p-Met and p-FAK protein levels determined via Western blot in 18 representative HCC tumor tissues **A.** and **B.** IHC in 2 paired patient sections.

The clinicopathologic significance of LCN2 in HCC and its correlation with TR were evaluated. LCN2 levels were analyzed in 80 consecutive HCC patients using qRT-PCR. Among the 80 HCC sample pairs, LCN2 was overexpressed in 71.3% (57 of 80) cancerous tissues, compared with matched noncancerous tissues, and TRα levels elevated by about 76.3% (61 of 80) in cancerous tissues. High TRα and LCN2 levels were commonly observed in patients with poor survival rates (Fig. 6A, left and middle panels). Linear regression analysis further revealed a significant positive correlation between LCN2 and TRα1 levels, based on the T/N ratio (Spearman correlation coefficien*t* = 0.3399, *P* = 0.002) (Fig. 6A, right panel). In contrast, no significant correlation between LCN2 and TRβ1 levels was evident (data not shown). We examined LCN2 and TRα1 expression in relation to several parameters. Both LCN2 and TRα displayed similar expression patterns relative to tumor grade (Fig. 6B), TNM stage (Figure 6C), and vascular invasion grade (Fig. 6D). Increased LCN2 expression and concomitantly elevated TRα, p-Met, and p-FAK levels in HCC tissues of 18 representative paired specimens are presented in Fig. 7A and 7B. Similar expression patterns of LCN2 and TRα were consistently observed in public Oncomine microarray datasets (Fig. S2). In conclusion, T_3_/TR-regulated LCN2 promotes cell migration and invasion via activation of the Met/FAK cascade and E-cadherin suppression. Our collective *in vitro* and *in vivo* findings support the potential utility of LCN2 as an effective therapeutic target for HCC treatment.

## DISCUSSION

In the current study, we showed that LCN2 is modulated by T_3_ at both mRNA and protein levels. Our experiments confirmed that T_3_ regulates *LCN2* at the transcriptional level, and TR proteins directly bind TRE between positions −1444 and −1427 of the *LCN2* 5′-flanking region. Notably, cell lines overexpressing LCN2 showed higher migration and invasion abilities, both *in vitro* and *in vivo*. Moreover, T_3_-mediated regulation of LCN2 occurred via the Met/FAK cascade and suppression of E-cadherin, leading to cancer cell progression. The potential mechanism of LCN2-induced EMT has been addressed (Fig. S4). According to previous findings, integrin α5β1 acts upstream of c-Met and promotes c-Met activation [24]. Here, we observed that overexpression of LCN2 induces upregulation of integrin α5β1, leading to the hypothesis that LCN2 activates c-Met/FAK through the integrin α5β1 pathway. In our experiments, c-Met/FAK was downregulated in integrin α5β1-depleted cells, compared with control cells (Fig. S4A). Moreover, EMT markers, slug and MMP9, displayed similar repression patterns after integrin α5β1 depletion. To further address whether slug and MMP9 act downstream of c-Met, experiments were performed with c-Met knockdown cell lines. Notably, slug and MMP9 levels were downregulated in c-Met-depleted cells, compared with control cells (Fig. S4B). The results collectively indicate that LCN2 induces EMT through the integrin α5β1/c-Met/FAK pathway (Fig. S3B).

Overexpression of LCN2 promotes mesenchymal-like cell morphology accompanied by loss of epithelial marker (E-cadherin) and increased expression of mesenchymal markers (vimentin, fibronectin and MMPs) that contribute to invasiveness [12, 13], which accounts for their roles in enhancing tumor cell motility for metastasis. In breast cancer, LCN2 is upregulated by the HER2/PI3K/AKT/NF-κB pathway. Conversely, decrease in LCN2 expression significantly reduces the invasion and migration abilities of HER2-positive breast cancer cells [25]. LCN2 has been shown to regulate the HIF-1α/VEGF cascade through Erk activation and enhance angiogenesis in the aggressive MDA-MB-231 cell line [26]. Knockdown of LCN2 suppresses the invasion of prostate cancer cells through downregulation of MMP-2 and MMP-9 [27]. Moreover, LCN2 is overexpressed in the intestine in colitis patients and acts as a negative prognostic indicator in colorectal cancer. However, several studies reported that LCN2 suppressed cell migration and invasion in colon cancer and in Ras-transformed mouse mammary cells [28]. LCN2 was also demonstrated to inhibit invasion and angiogenesis in pancreatic cancer [29]. In Wang's study [30], LCN2 negatively modulated the HCC cells through an EGF (or TGF-β1)/LCN2/Twist1 pathway. According to their clinical data, 62.5% (25 of 40) HCC specimens expressed higher levels of LCN2 mRNA than adjacent nontumor liver tissue samples. Levels of LCN2 mRNA and protein significantly increased in the differentiation status of HCCs. Immunohistochemical staining in their cohort of patients demonstrated that LCN2 expression also increased in various tumor stages. Their results suggested that LCN2 expression is significantly correlated with a worse differentiation grade, but negatively correlated with twist1 in HCC. Collectively, LCN2 showed the similar expression pattern at HCC specimens in our results, but the regulation pathway and cell type are different. In our study, overexpression of LCN2 led to suppression of E-cadherin and increase in p-Met and p-FAK protein levels, while its depletion rescued E-cadherin and suppressed p-Met and p-FAK protein levels as well as MMP-9 and MMP-2 activities.

E-cadherin is a transmembrane protein that participates in rearrangement of the cytoskeleton and cell-cell junctions in cancer cell progression [31]. E-cadherin overexpression attenuates tumor cell migration and metastasis [32, 33]. In view of our finding that expression of E-cadherin is decreased in LCN2-overexpressing cells, compared with controls, and conversely rescued in LCN2-depleted cells, we propose that LCN2 accelerates tumor cell migration through alterations in E-cadherin expression.

c-Met is a receptor tyrosine kinase that binds and interacts with its ligand, hepatocyte growth factor (HGF), to activate different cellular signaling pathways, including proliferation, motility, migration and invasion [34]. Cell migration is mediated downstream of c-Met through phosphorylation of focal adhesion kinase (FAK), which is localized to cellular adhesion complexes. A recent study demonstrated that Met-FAK interactions are a critical determinant for tumor cells to acquire invasive potential [23]. Our study disclosed that phosphorylation of c-Met (Y1349) and FAK (Y397) is increased in LCN2-overexpressing cells, compared with controls. Conversely, phosphorylation is decreased in LCN2-depleted cells. Notably, however, HGF mRNA and protein levels were not significantly changed upon LCN2 overexpression or knockdown (data not shown). These findings indicate that LCN2 promotes c-Met, but not a ligand-dependent activation pathway. We propose that LCN2 regulates tumor cell migration through activation of the Met/FAK cascade.

The thyroid hormone is a critical regulator of diverse cell functions. TH participates in cell proliferation, metabolism, organ development and muscle control in a normal physiological environment [35, 36]. T_3_/TR stimulates or inhibits the expression of suppressor genes-DKK4 [37] or oncogenes-PTTG1 [16], respectively in a subset of HCC specimens. During cancer progression, T_3_/TR may play dual roles to promote or suppress cancer cells in a certain genetic background, similar to the TGF-βpathway [33]. Specifically, in benign tumors or early-stage cancer, T_3_/TR may inhibit cancer cell proliferation, but promote cancer cell migration and invasion in malignant tumors or late-stage cancer. Recent studies indicate that T_3_/TR signaling upregulates proteases, such as cathepsin H (CTSH) [38]. and brain-specific serine protease 4 (BSSP4) [39] for ECM cleavage, and promotes cancer metastasis, supporting a potential oncogenic role. However, the function of T_3_/TR signaling in tumorigenesis cannot be prematurely established from a single gene study, and “the whole genome T_3_ effect” must be considered. Establishing the effect of T_3_/TR on an animal model is a plausible means to define its role in cancer. Since TR actions are complex, tissue- and time-specific aberrant expression patterns of various TR isoforms have different effects and are associated with different tumor types or stages of development. T_3_ signaling and its interactions with other coregulators may facilitate the switch from tumor suppression in the premalignant stages of tumorigenesis to promotion in later stages of liver cancer, leading to metastasis. Further studies are necessary to determine what conditions that TRs might act as a tumor suppressor and in what other conditions, TRs could play as an oncogene. Data from our current study showed that T_3_/TR modulates LCN2, a cancer-associated protein, to promote cancer progression.

In conclusion, LCN2 enhances migration and invasion abilities in HCC cell lines, both *in vitro* and *in vivo*. T_3_-regulated LCN2 may play a role in hepatoma cell motility through activation of the Met/FAK cascade to promote metastasis.

## SUPPLEMENTARY FIGURES


