# Supplementary material for: Thyroid hormone-mediated regulation of lipocalin 2 through the Met/FAK pathway in liver cancer
Source: Oncotarget. 2015 Apr 10;6(17):15050–64. doi: 10.18632/oncotarget.3670 (PMC4558135; doi:10.18632/oncotarget.3670)
Supplement: Supplementary file 1 [file oncotarget-06-15050-s001.pdf]

SUPPLEMENTARY FIGURES

(A)

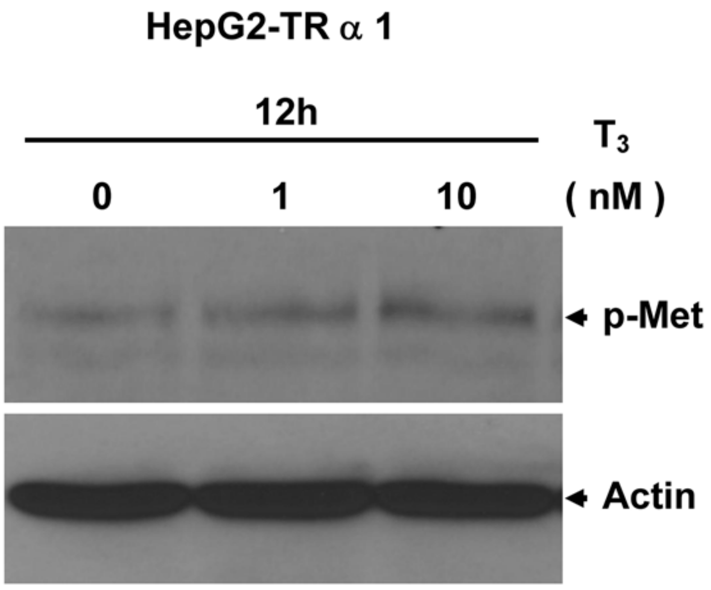

(B)

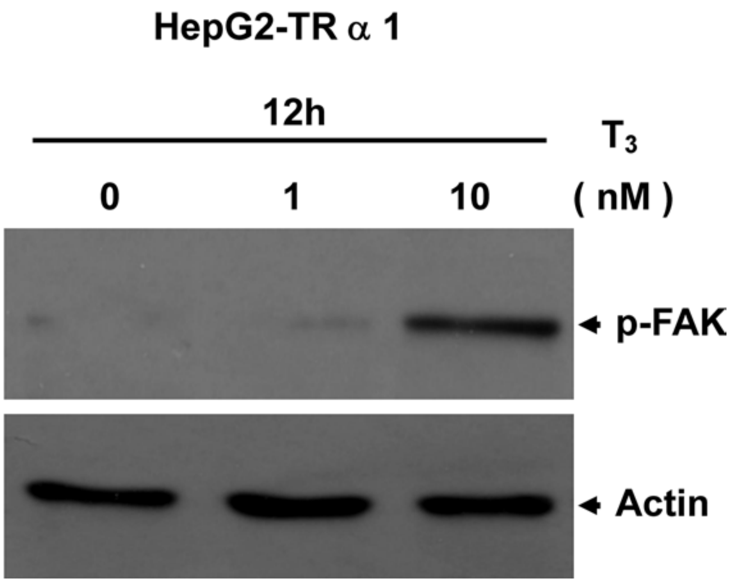

Supplementary Figure S1: T_3 regulates p-Met and p-FAK in HepG2-TRα1 cells. Expression levels of **A.** p-Met and **B.** p-FAK in TRα1-overexpressing cells were determined via Western blot after 0, 1 or 10 nM T_3 treatment for 12 h.

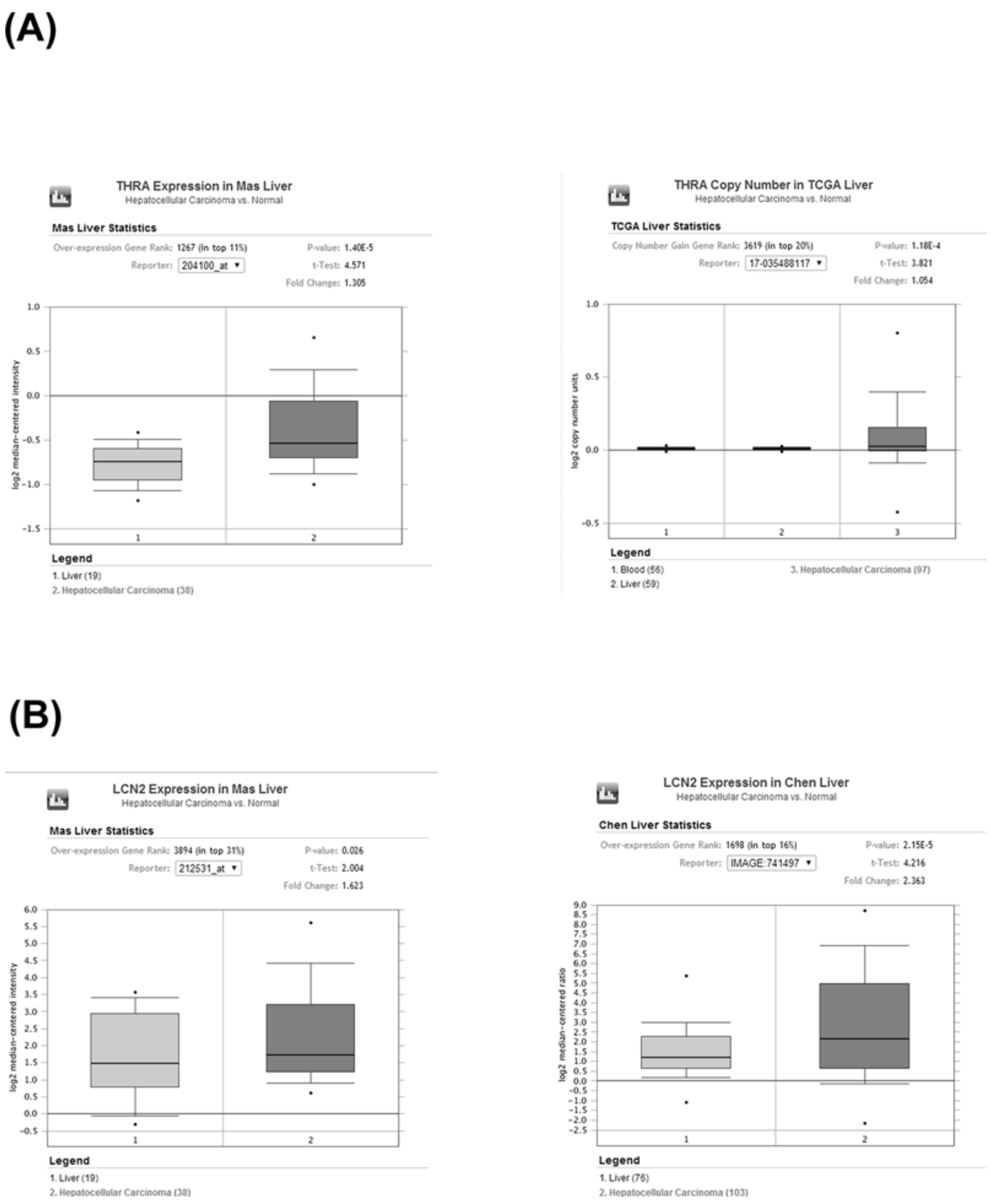

Supplementary Figure S2: Expression of LCN2 and TRα in public Oncomine microarray datasets. In the two Oncomine datasets (Mas and TCGA), TRα was highly expressed **A.** with a higher copy number in HCC **B.**, compared to normal cells (liver). Similar high LCN2 expression was observed in both Mas and Chen HCC datasets.

(A)

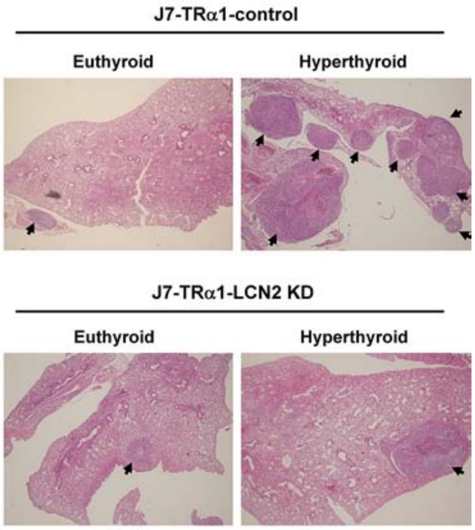

(B)

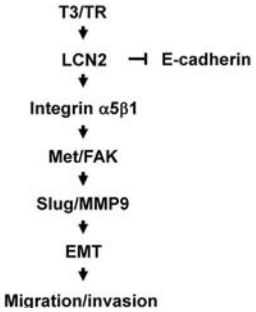

Supplementary Figure S3: *In vivo* model of T3-induced cell invasion under LCN2 depletion. A. The images depict lung tumor foci of mice injected with J7-TRα1-control (Luc) and J7-TRα1-LCN2 KD cells, and administered T_3 (2 mg/L) in drinking water for hyperthyroid condition (right panel), compared with euthyroid (left panel). The metastatic foci in lung for J7-TRα1-control and J7-TRα1-LCN2 KD cell are indicated by arrowhead and staining with H&E. **B.** Schematic diagram showing that T_3/TR-regulated LCN2 promotes hepatoma cell migration through activation of the Integrin α5β1/Met/FAK cascade and modulation of EMT markers, such as slug and MMP-9.

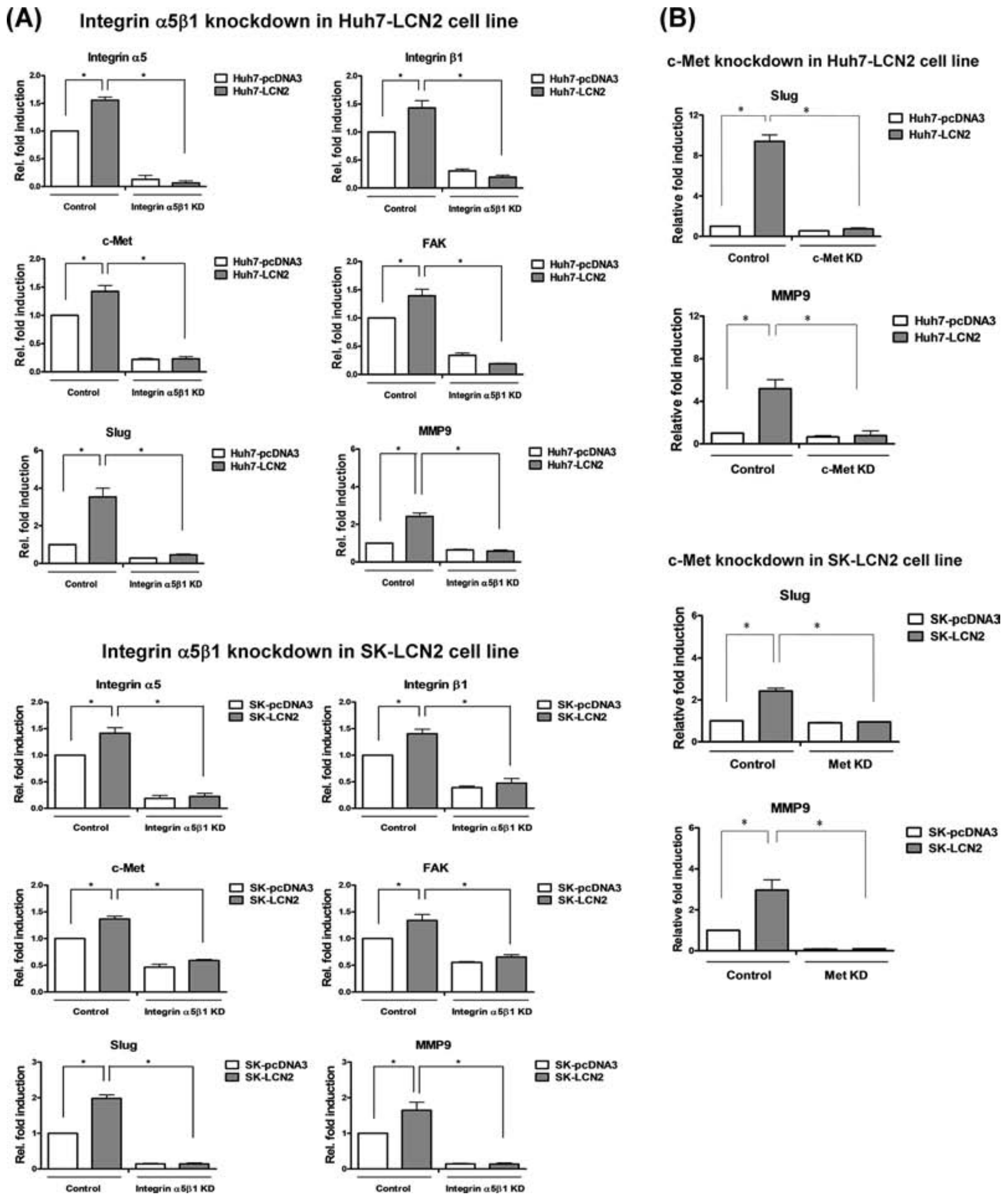

Supplementary Figure S4: Effect of Integrin α5β1 or c-Met-depletion on LCN2 overexpression cell line. A. The mRNA expression level of Integrin α5, Integrin β1, c-Met, FAK, slug and MMP9 was detected in integrin α5β1-depleted Huh7-pcDNA 3.0 and Huh7-LCN2 cells, compared with control cells (upper panel). Similar results were observed in Integrin α5β1-depleted SK-pcDNA 3.0 and SK-LCN2 cells (lower panel). **B.** The mRNA expression level of slug and MMP9 was detected in c-Met-depleted Huh7-pcDNA 3.0 and Huh7-LCN2 cells, compared with control cells (upper panel). Similar results were observed in c-Met depleted SK-pcDNA 3.0 and SK-LCN2 cells (lower panel).

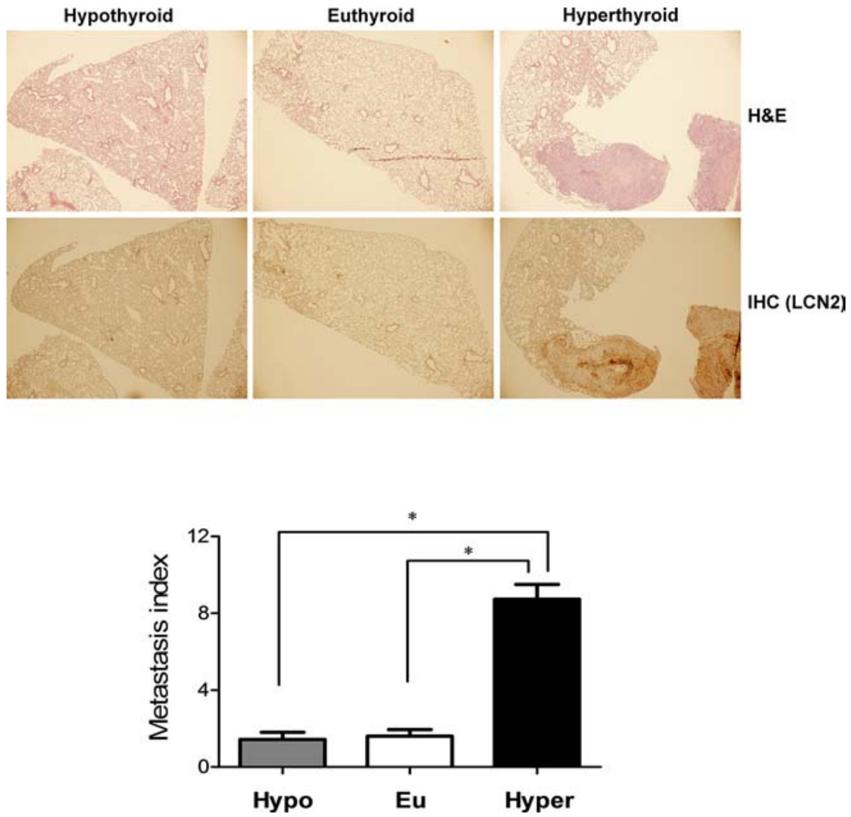

Supplementary Figure S5: Effect of T3 on LCN2-mediated cell migration and invasion. The tumor foci of lung of SCID mice in the hypothyroid, euthyroid and hyperthyroid condition were analyzed with H&E (upper panel) and IHC (lower panel) staining to examine LCN2 expression and tumor foci. The numbers of tumor foci from at least five areas were calculated as a Metastasis index. Differences were analyzed using One-way ANOVA, $*P < 0.05$.
